# VEGF Stimulates Activation of ERK5 in the Absence of C-Terminal Phosphorylation Preventing Nuclear Localization and Facilitating AKT Activation in Endothelial Cells

**DOI:** 10.3390/cells12060967

**Published:** 2023-03-22

**Authors:** Anil Kumar Mondru, Mohammad A. Aljasir, Ahmed Alrumayh, Gopika N. Nithianandarajah, Katie Ahmed, Jurgen Muller, Christopher E. P. Goldring, Bettina Wilm, Michael J. Cross

**Affiliations:** 1 Department of Pharmacology and Therapeutics, Institute of Systems, Molecular and Integrative Biology, University of Liverpool, Liverpool L69 3GE, UK; 2 Cardiovascular Research Group, School of Pharmacy and Medical Sciences, University of Bradford, Bradford BD7 1DP, UK; 3 Department of Molecular Physiology and Cell Signalling, Institute of Systems, Molecular and Integrative Biology, University of Liverpool, Liverpool L69 3BX, UK

**Keywords:** angiogenesis, ERK5, EGF, EGFR, VEGF-A, VEGFR-2, AKT, endothelial cells

## Abstract

Extracellular-signal-regulated kinase 5 (ERK5) is critical for normal cardiovascular development. Previous studies have defined a canonical pathway for ERK5 activation, showing that ligand stimulation leads to MEK5 activation resulting in dual phosphorylation of ERK5 on Thr218/Tyr220 residues within the activation loop. ERK5 then undergoes a conformational change, facilitating phosphorylation on residues in the C-terminal domain and translocation to the nucleus where it regulates MEF2 transcriptional activity. Our previous research into the importance of ERK5 in endothelial cells highlighted its role in VEGF-mediated tubular morphogenesis and cell survival, suggesting that ERK5 played a unique role in endothelial cells. Our current data show that in contrast to EGF-stimulated HeLa cells, VEGF-mediated ERK5 activation in human dermal microvascular endothelial cells (HDMECs) does not result in C-terminal phosphorylation of ERK5 and translocation to the nucleus, but instead to a more plasma membrane/cytoplasmic localisation. Furthermore, the use of small-molecule inhibitors to MEK5 and ERK5 shows that instead of regulating MEF2 activity, VEGF-mediated ERK5 is important for regulating AKT activity. Our data define a novel pathway for ERK5 activation in endothelial cells leading to cell survival.

## 1. Introduction

Mitogen-activated protein kinases (MAPKs) are enzymes that exist in a three-tier modular cascade, with the MAPK kinase kinase (MAPKKK) activating MAPK kinase (MAPKK) and terminating with activation of the MAPK. The sequential phosphorylation of the MAPK module is initiated by extracellular stimuli such as cytokines, environmental stresses, growth factors, and other stimuli [1,2]. Once the final MAPK is activated, it is able to elicit specific intracellular responses via phosphorylation and modification of its downstream effector molecules, which include transcription factors, cytoplasmic enzymes and structural proteins [3,4,5,6].

Extracellular-signal-regulated kinase 5 (ERK5), also known as big mitogen-activated protein kinase-1 (BMK1), exists downstream of a linear signaling cascade consisting of MEKK2, MEKK3, and MEK5 [7,8] (reviewed in [9,10]). A multitude of extracellular stimuli, including oxidative and osmotic stresses [11], vascular endothelial growth factor (VEGF), epidermal growth factor (EGF) [12], brain-derived neurotrophic factor [13], specific inflammatory cytokines such as interleukin 6 (IL-6) [14], as well as ischaemic and hypoxic conditions [15,16], activate MEKK2 and MEKK3, which, in turn, phosphorylate MEK5 on Ser311/Thr315 [17,18]. As the only upstream MAPKK to activate ERK5, MEK5 dual-phosphorylates the Thr218/Tyr220 residues of the T-E-Y motif contained within the activation loop [19], thereby enabling ERK5 to autophosphorylate several residues on its uniquely extended C-terminal tail and enhancing the transcriptional activity of ERK5 [20].

In its unphosphorylated state, it has been proposed that ERK5 exists in a folded conformation, sequestered in the cytoplasm [21,22]. It is considered that this folded conformation may either dampen the nuclear localisation signal (NLS), or generate a nuclear export signal (NES) due to an intramolecular interaction between the N- and C-terminals. Upon dual phosphorylation, however, a conformational change occurs to ERK5, whereby the presumed NES interaction is disrupted, resulting in ERK5 translocation to the nucleus [23]. Following nuclear translocation, ERK5 phosphorylates members of the myocyte enhancer factor-2 (MEF2) family of transcription factors leading to MEF2-dependent gene expression [12].

The important physiological role of ERK5 was highlighted when it was shown that *Erk5*-deficient mice died at embryonic day 10.5 (E10.5) as a consequence of severe defects to normal angiogenesis and the maturation of vasculature [16,24,25,26]. Importantly, targeted deletion of *Erk5* in murine endothelial cells, but not in other cell types such as cardiomyocytes and hepatocytes, was also found to result in cardiovascular defects and disrupted vascular integrity, a phenotype that is identical to that of the global *Erk5* knockout mouse [26,27]. These data suggest that although ERK5 is expressed in a variety of tissues, expression of ERK5 within the endothelium is crucial to preserve vascular integrity. Our previous data have shown that in human dermal microvascular endothelial cells (HDMECs), VEGF stimulation of ERK5 is required for AKT activation and suppression of apoptosis leading to tubular morphogenesis of endothelial cells [28]. This suggests that ERK5 may perform a unique function in endothelial cells relative to other cells. In our current study, we analysed VEGF-mediated activation of ERK5 in HDMECs and compared this with EGF-mediated ERK5 activation in HeLa cells. Our data show that in HDMECs, the VEGF/VEGFR-2 signalling axis activates ERK5 in the absence of C-terminal phosphorylation, which prevents nuclear translocation of ERK5 and subsequent regulation of MEF2-mediated gene expression. Instead, we found that following VEGF stimulation, ERK5 is required for efficient AKT phosphorylation and co-localises with AKT in a cytoplasmic and plasma membrane location. Overall, our data show that VEGFR-2 couples to ERK5 in endothelial cells via a unique mechanism to regulate endothelial cell survival.

## 2. Materials and Methods

### 2.1. Agonists and Inhibitors

Recombinant human vascular endothelial growth factor (VEGF)-A165 was purchased from R&D Systems Inc. (Abingdon, UK). Recombinant human epidermal growth factor (EGF) was purchased from Peprotech (London, UK). BIX02189, AX15836 and BIRB796 were purchased from Tocris Bioscience (Bristol, UK), and JWG071 was purchased from Sigma (Poole, UK).

### 2.2. Antibodies and Reagents

Antibodies against total ERK5 (#12950), MEK5 (#91670), p38 MAPK (#8690), Actin (#8456), EGFR1 (#4267) and VEGFR2 (#2479) AKT (#4691), p44/42 MAPK (#4696), MAPKAPK2 (#3042) and antibodies against phospho-AKT S473 (#4060 and Alexa Fluor^®^ 488 Conjugate (#2336), phospho-p44/42 MAPK T202/Y204 (#4370), phospho-ERK5 T218/Y220 (#3371), phospho-EGFR1 Y1061 (#3777), phospho-VEGFR2 Y1175 (#3770), phospho-p38 MAPK T180/Y182 (#4631), phospho-MAPKAPK2 Thr334 (#3007) were purchased from Cell Signalling Technology (Leiden, Netherlands). Antibodies against MEKK2 (#ab33918) and MEKK3 (#ab40750) were purchased from Abcam (Cambridge, UK). All commercial antibodies were used at a dilution of 1:1000 for Western blotting. Phospho-specific antibodies against T218/Y220 and T732 ERK5 were generated and kindly supplied by Dr. Atanasio Pandiella (Instituto de Biolgia Molecular y Celular del Cancer, Salamanca, Spain). Horseradish peroxidase (HRP)-conjugated AffiniPure goat anti-rabbit IgG (H+L) secondary antibody (#111-035-144) was purchased from Jackson ImmunoResearch Laboratories, Inc. (West Grove, PA, USA). Alexa Fluor^®^ 488 donkey anti-rabbit IgG (H+L) (#A21206) and Hoechst 33342 (H21492) were purchased from Molecular Probes^®^ (Life Technologies™; Paisley, UK). Enhanced chemiluminescence (ECL) substrate for detection of HRP was bought from Thermo Fisher Scientific™ (Waltham, MA, USA). All other chemicals and reagents were obtained from Sigma-Aldrich (Poole, UK) unless otherwise indicated.

### 2.3. Cell Culture

Human dermal microvascular endothelial cells (HDMECs, #C-12210) and human cardiac microvascular endothelial cells (HCMECs, #C-12285) were obtained from Promocell (Heidelberg, Germany). Cells were grown in Endothelial Cell Growth Medium MV2 (ECGM-MV2) containing Endothelial Cell Basal Medium MV2 (ECBM-MV2) supplemented with 5% FCS, 5 ng/mL recombinant Human Epidermal Growth Factor, 10 ng/mL bFGF, 20 ng/mL Long R3 Insulin-like Growth Factor, 0.5 ng/mL recombinant human Vascular Endothelial Growth Factor A 165 (VEGF-A_165_), 1 μg/mL ascorbic acid, and 0.2 μg/mL hydrocortisone (#C-22121, Promocell, Heidelberg, Germany) and Penicillin/Streptomycin. Cells were routinely cultured on 0.5% (*w/v*) gelatin-coated plates (G1890, Sigma) and grown between passages 2-8. Human epithelial cervical cancer cell line (HeLa) were obtained from the American Type Culture Collection (ATCC). Cells were cultured in Dulbecco’s Modified Eagle Medium (DMEM; #11885084) supplemented with 10% (*v/v*) FCS and Penicillin/Streptomycin. For serum starvation, cells were incubated overnight in DMEM supplemented with 1% (*v/v*) FCS. Murine aortic endothelial (MAE) cells and MAE cells expressing Flk-1 were kindly supplied by Prof. Lena Claesson-Welsh (Department of Immunology, Genetics and Pathology, Uppsala University, Uppsala, Sweden). All MAE cells were cultured in DMEM supplemented with 10% (*v/v*) FCS containing Penicillin/Streptomycin and serum-starved in DMEM supplemented with 1% (*v/v*) FCS. All cells were cultured at 37 °C in a humidified 5% CO_2_ atmosphere.

### 2.4. siRNA Knockdown

SiRNA duplexes were prepared as per the manufacturer’s instructions. Non-silencing #D-001810-10-05, #MEKK2 L-003582-02-0005, #MEKK3 L-003301-00-0005, #MEK5 L-003966-00-0005, and #ERK5 L-003513-00-0005 smartpools were purchased from Dharmacon (Horizon Discovery, Cambridge, UK).

Cells were plated in 12-well plates (5.0 × 10^4^ HDMECs or 6.5 × 10^4^ HeLa cells per well) and allowed to grow for 24 hours, ensuring 80% confluency. Fresh culture medium was added to the cells which were then transfected with a final concentration of 10 nM siRNA in OptiMEM (Life Technologies, Paisley, UK) and 0.2% Lipofectamine RNAiMAX (Life Technologies, Paisley, UK). Cells were incubated with the transfection mix for 6 h at 37 °C, then washed twice with PBS and incubated with appropriate fresh culture medium for an additional 24 h at 37 °C. Cells were washed with PBS and serum-starved overnight in their appropriate low-serum media prior to agonist stimulation and cell lysis. 

### 2.5. Agonist Stimulation

All growth factors were initially diluted in sterile-filtered PBS containing 0.1% (*w/v*) BSA to 100 μg/mL stock concentrations. For experimental use, growth factors were diluted in appropriate low-serum medium to 50 ng/mL final concentration. After overnight serum starvation, cells were incubated with different agonists for the required time period, prior to cell lysis.

### 2.6. RIPA Protein Extraction and Western Blot

After all cell treatments, cells were washed twice with ice-cold PBS then lysed with modified radio-immunoprecipitation assay (RIPA) lysis buffer (20 mM Tris-HCl; pH 7.5, 150 mM NaCl, 2.5 mM EDTA, 10% (*v/v*) glycerol, 1% (*v/v*) Triton-X-100, 1 mM Na_3_VO_4_, 10 μg/mL aprotinin, 10 μg/mL leupeptin, 10 μg/mL pepstatin, 1 mM phenylmethylsulphonyl fluoride (PMSF), 0.1% (*w/v*) SDS and 0.5% (*w/v*) sodium deoxycholate. Cell lysates were centrifuged (14,000 rpm for 20 min at 4 °C) and protein levels determined by BCA assay (Pierce/Thermo Fisher #23227). Lysates were subsequently mixed with 4X LDS sample buffer, boiled at 90 °C for 5 min, centrifuged for 30 s and stored at −80 °C. Samples containing 20 μg of protein were then subjected to conventional SDS-PAGE. Ten% acrylamide Tris-Glycine gels were generally used, with 7.5% acrylamide Tris-Glycine gels used for ERK5 analysis. Following electrophoresis, gels were transferred to nitrocellulose membranes (Hybond C, GE Healthcare, Chicago, IL, USA), and blocked with 5% (*w/v*) Bovine Serum Albumin Fraction V (BSA; 10735108001, Merck, Rahway, NJ, USA) in Tris-buffered saline (pH 7.6). Blots were probed with primary antibodies and later HRP-coupled secondary antibodies, both diluted in TBS containing 0.1% (*v/v*) Tween 20 and 2% (*w/v*) BSA. Chemiluminescence was detected following incubation with Pierce™ ECL Western blotting (32106, Thermo Fisher, Waltham, MA, USA) and exposure to photographic film (Fuji Medical X-Ray Film, Super RX, 100NF; Jet X-Ray, London, UK). Digitalized grayscale images were used for optical density measurement by using ImageJ software (National Institute of Health (NIH). Density values were normalised to actin levels measured in the same membrane.

For Phos-tag gels, cells were lysed in RIPA buffer-containing protease inhibitors without EDTA. Phos-tag™ reagent (#304-93521) was purchased from Alpha Labs (Hampshire, UK) and prepared according to the manufacturer’s instructions and as previously described [29], to give a 5 mM stock solution in water/methanol. Phos-tag gels were prepared using 7.5% (*w/v*) acrylamide containing 40 μM Phos-tag and 80 μM MnCl_2_. Gels were run at 30 mA for 15 h. Prior to protein transfer, Phos-tag gels were incubated and gently agitated in distilled water for 5 min. All membranes were then immunoblotted with the desired antibodies. To re-probe a blot, the membrane was stripped by incubating for 30 min at 50 °C in stripping buffer (0.5M Tris-HCl (pH 6.8), 4% SDS, 100 mM β-mercaptoethanol), washed three times in Tris-buffered saline (TBS; 20 mM Tris, 0.137 M NaCl) containing 0.1% (*v/v*) Tween-20 (TBS-T), and incubated in blocking buffer (TBS-T containing 5% (*w/v*) bovine serum albumin (BSA)).

### 2.7. Immunofluorescence

3.0 × 10^4^ HDMECs and 4.0 × 10^4^ HeLa cells were seeded onto sterile 16 mm glass coverslips placed within wells of a 12-well plate in their appropriate culture medium for 48 h at 37 °C. Cells were then serum-starved overnight in their appropriate low-serum media, prior to agonist stimulation and washed with ice-cold PBS. Ice-cold methanol was then added to the cells for 10 min and incubated at −20 °C for cell fixation and permeabilisation, after which cells were washed thrice with TBS containing 0.1% (*v/v*) Tween-20 (TBS-T). Cells were blocked with 1% (*w/v*) BSA, 5% (*v/v*) donkey serum (#D9663, Sigma), TBS-T for 30 min. Cells were immunostained with anti-ERK5 antibody (#3372 CST, Danvers, MA, USA) diluted 1:100 in 1% (*w/v*) BSA, TBS-T for 2 h under shaking conditions at room temperature, followed by an incubation with donkey anti-rabbit Alexa568 secondary antibody (#A-10042 Thermo Fisher) diluted 1:1000 in 1% (*w/v*) BSA, TBS-T for 2 h under mild shaking conditions, at room temperature and covered from the light. Coverslips were washed in TBS-T and incubated with anti-phospho-Akt Ser473-Alexa488 conjugate (#2336, Thermo Fisher) diluted 1:100 for 1 hour under mild shaking at room temperature and protected from the light. Coverslips were finally incubated with 2 μg/mL Hoechst 33342 (#H21492, Thermo Fisher) and mounted on microscope slides using ProLong™ Gold Antifade Mountant (#P36930, Thermo Fisher), sealed, and stored at 4 °C until imaging. Images were obtained using a Zeiss AxioObserver Z1 (Zeiss, Cambridge, UK) epifluorescent inverted microscope with Apotome2 and acquired using ZenPro 3.3 software and imaged using a Plan-Apochromat 40×/1.2 oil-immersion objective. Z-stack images were obtained for each sample and a maximum intensity projection (MIP) generated.

### 2.8. qRT-PCR 

Total RNA was purified from HDMECs and HeLa cells using the RNeasy kit, according to the manufacturer’s instructions (Qiagen, Manchester, UK). RNA quantity and quality were determined using a NanoDrop 1000 Spectrometer (Thermo Fisher Scientific). Then, 1 µg of RNA was reverse-transcribed to cDNA using the reverse transcriptase Superscript III in addition to oligo dT, random hexamers, dNTP mix, and RiboLock (Life Technologies, Paisley, UK). To set up a 25 µL reaction for qPCR, 10ng of cDNA was mixed with RNase free water, 2× Power SYBR Green (Life Technologies, Paisley, UK) and forward and reverse primers to a final concentration of 400 nM (Life Technologies, Paisley, UK). 

Primer sequences (5’ to 3’): 

*β-ACTIN* Fwd GATGAGATTGGCATGGCTTT; *β-ACTIN* Rev CACCTTCACCGTTCCAGTTT. *MEF2A* Fwd CAGGGAGTTCACTGGTGTCC; *MEF2A* Rev CTTGGTGGTCTCTGAGGAGC. *MEF2B* Fwd GTTCACCAAGCGGAAGTTCG; *MEF2B* Rev GCATACTGGAAGAGGCGGTT. *MEF2C* Fwd CGAGATGCCAGTCTCCATCC; *MEF2C* Rev GTGAGCCAGTGGCAATAGGT. *MEF2D* Fwd AGGAAGAAGGGCTTCAACGG; *MEF2D* Rev GTCGGTACTTGTCCTCCAGC.

qPCR was performed on an ABI ViiA 7 Thermocycler (Applied Biosystems) with the following parameters: 50 °C for 2 min; 95 °C for 10 min; (95 °C for 15 s and 60 °C for 1 min) × 40 cycles. For each sample, the average cycle threshold (CT) value was normalised to *β-ACTIN* and then compared to the relevant control sample using the comparative CT (2-ΔΔCT) method. 

### 2.9. MEF2 Reporter Assay

Ad-MEF2-Luc was kindly provided by Dr. Alessandro Cannavo (Temple University, Philadelphia, PA, USA) and Dr. Jeff Molkentin (Howard Hughes Medical Institute, Cincinnati, OH, USA). Ad-CMV-LacZ was kindly provided by Prof. Beverley Rothermel (University of Texas Southwestern Medical Center, Dallas, USA). Reporter lysis buffer and Bright-GloTM Luciferase system were purchased from Promega, Southampton, UK. ONPG substrate powder was purchased from Thermo Fisher Scientific, UK.

For MEF2 reporter assays, HDMECs (0.45 × 10^5^) and HeLa (0.7 × 10^5^) cells per well were seeded into 12-well plates for 24 h. The following day, cells were transduced with Ad-MEF2-Luc multiplicity of infection (MOI; ratio of infectious units to the number of cells) of 50 and Ad-CMV-LacZ (MOI 20) in full growth medium or complete medium for 24 h. Cells were then serum-starved overnight and treated with either vehicle control (0.1% DMSO) or the MEK5 inhibitor BIX02189 (BIX; 1 μM), or the p38 MAPK inhibitor BIRB796 (BIRB; 1 μM), for 1 h prior to stimulation with VEGF (50 ng/mL) or EGF (50 ng/mL) for 6 h. Following stimulation, cells were then washed in PBS, lysed in 5x reporter lysis buffer (#E3971 Promega), and centrifuged. The supernatant was then mixed with luciferase assay substrate (#E2610 Promega) and reporter activity determined by measuring luminescence using a Varioskan plate reader. As an internal control, β-galactosidase activity was measured to normalise for transfection efficiency.

### 2.10. Statistical Analysis

Data are presented as mean ± SEM (*n* = 3). Statistical analysis was performed using GraphPad Prism 9 software with comparisons performed using one-way ANOVA followed by Tukey’s post hoc test; * *p* < 0.05, ** *p* < 0.01 and *** *p* < 0.001. In all cases, the confidence interval was set at 95%, and statistical significance was set at *p* < 0.05.

## 3. Results

### 3.1. VEGF-Stimulated ERK5 Fails to Undergo C-Terminal Phosphorylation

ERK5 is ubiquitously expressed in cells. However, the defective vascular phenotype of the ERK5^−/−^ knockout mouse leading to embryonic lethality suggests that ERK5 may play a unique role in endothelial cells [30]. Following agonist-mediated stimulation of ERK5 on Tyr218/Thr220 in the activation loop, ERK5 is known to undergo autophosphorylation leading to phosphorylation of C-terminal residues on Ser and Thr [31] and nuclear translocation of the protein [32]. Activation of ERK5 by agonists such as EGF in HeLa cells has been shown to stimulate a band shift in ERK5 detectable using normal SDS-PAGE followed by Western blotting [11,33,34]. However, we noted in the course of our studies using endothelial cells, that whilst VEGF stimulated phosphorylation of ERK5 on Tyr218/Thr220, a band shift was not evident using conventional SDS-PAGE [28].

**Figure 1:**
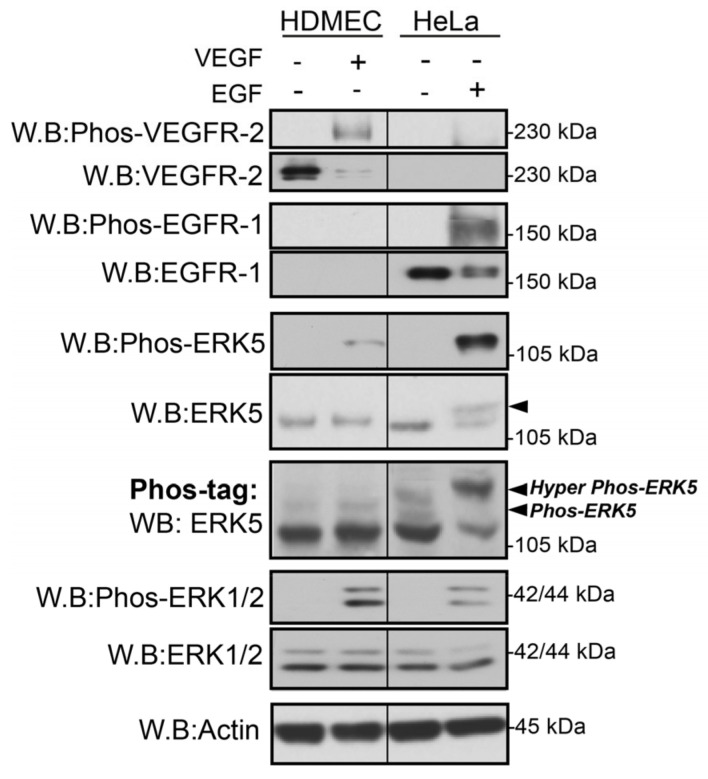
**VEGF and EGF stimulate differential ERK5 phosphorylation**. HDMECs and HeLa cells were serum-starved overnight prior to stimulation for 10 min with either VEGF (50 ng/mL) or EGF (50 ng/mL), respectively. Cells were lysed in RIPA buffer, and lysates were resolved by 7.5–10% SDS-PAGE or Phos-tag SDS-PAGE and analysed by Western blotting. Results are from one experiment representative of three independent experiments.

We have recently utilised a novel sensitive assay to analyse VEGF-mediated ERK5 phosphorylation based on Phos-tag acrylamide, which retards the phosphorylated ERK5, allowing efficient separation from unphosphorylated ERK5 [29]. Phos-tag acrylamide allows simultaneous discrimination of multiple phosphoproteins based on differential mobility shift by SDS-PAGE [35]. Using this experimental approach, we aimed to determine if VEGF-stimulated ERK5 undergoes C-terminal phosphorylation in HDMECs. As a control, we utilized the HeLa cervical cancer cell line stimulated with EGF, which is known to induce C-terminal phosphorylation of ERK5 [31]. HeLa cells were stimulated with EGF (50 ng/mL) and HDMECs with VEGF (50 ng/mL) for 10 min, and protein lysates were obtained, before being separated on a conventional SDS PAGE Gel and a Phos-tag gel (Figure 1). Analysis of the gels revealed that VEGF and EGF stimulated phosphorylation of ERK5 and ERK1/2 using phospho-antibodies to the TEY motif. However, using conventional SDS-PAGE, only EGF was able to induce a band shift, with VEGF failing to induce any discernable shift. The Phos-tag gel revealed that VEGF was able to evoke a band shift in HDMECs (labelled Phos-ERK5), but not to the same degree as EGF stimulation of HeLa, where a much larger band shift was observed, suggesting phosphorylation of ERK5 on multiple residues following EGF stimulation (labelled Hyper Phos-ERK5; Figure 1).

**Figure 2:**
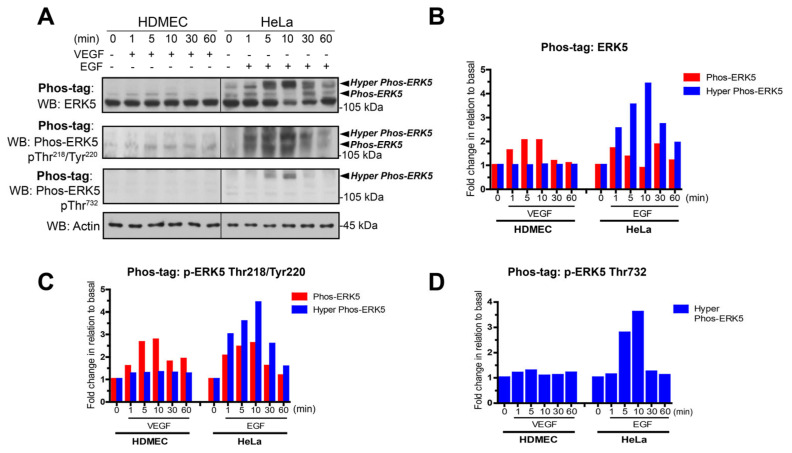
VEGF does not stimulate C-terminal phosphorylation of ERK5 in HDMECs. (**A**) HDMECs and HeLa cells were serum-starved overnight prior to stimulation for various time periods with either VEGF (50 ng/mL) or EGF (50 ng/mL), respectively. Cells were lysed in RIPA buffer and lysates were resolved by Phos-tag SDS-PAGE and analysed for ERK5 activation using site-specific phospho-antibodies. Protein loading was confirmed by blotting for Actin. (**B**) Quantification of ERK5 level by densitometric analysis. Data are shown as mean normalised to Actin level (*n* = 2). (**C**) Quantification of phospho-ERK5 (T218/Tyr220) level by densitometric analysis. Data are shown as mean normalised to Actin level (*n* = 2). (**D**) Quantification of phospho-ERK5 (Thr732) level by densitometric analysis. Data are shown as mean normalised to Actin level (*n* = 2).

A similar differential pattern of VEGF-stimulated ERK5 activation was shown in HDMECs obtained from two independent donors and also in human cardiac microvascular endothelial cells (HCMECs) (Supplementary Figure S1). We analysed this potential differential phosphorylation between VEGF stimulation in HDMECs and EGF stimulation of HeLa cells over a range of different time points (Figure 2). The Phos-tag gels probed with ERK5 antibody revealed that VEGF stimulated a band shift with only one retarded band evident at approximately 115 kDa, which was maximal at 10 min but sustained for 60 min. In contrast, in HeLa cells, EGF stimulated a band shift with a number of distinct retarded bands evident at approximately 115 kDa and 125–130 kDa. This suggested that a number of phosphorylation events were occurring with EGF stimulation that were not evident with VEGF. To analyse the phosphorylation events, the blots were stripped and reprobed with an antibody to phospho-Tyr218/Thr220 in the activation loop and phospho-Thr732 in the C-terminal tail [36]. It was clear that in the HDMECs, VEGF stimulated phosphorylation on Thr218/Tyr220 with the band migrating at approximately 115 kDa; however, no phosphorylation on Thr 732 was evident. In contrast, in EGF-stimulated HeLa cells probed with phospho-Thr218/Tyr220 antibody, two bands were evident at approximately 115 kDa and 125 kDa. When probed with phospho-Thr732 antibody, only one band was evident at approximately 125 kDa. Taken together, these data suggest that, whilst stimulation with VEGF or EGF results in phosphorylation of Thr218/Tyr220 in the N-terminal activation loop, unlike EGF, VEGF stimulation fails to subsequently induce phosphorylation of Thr732 in the C-terminal tail of ERK5.

**Figure 3:**
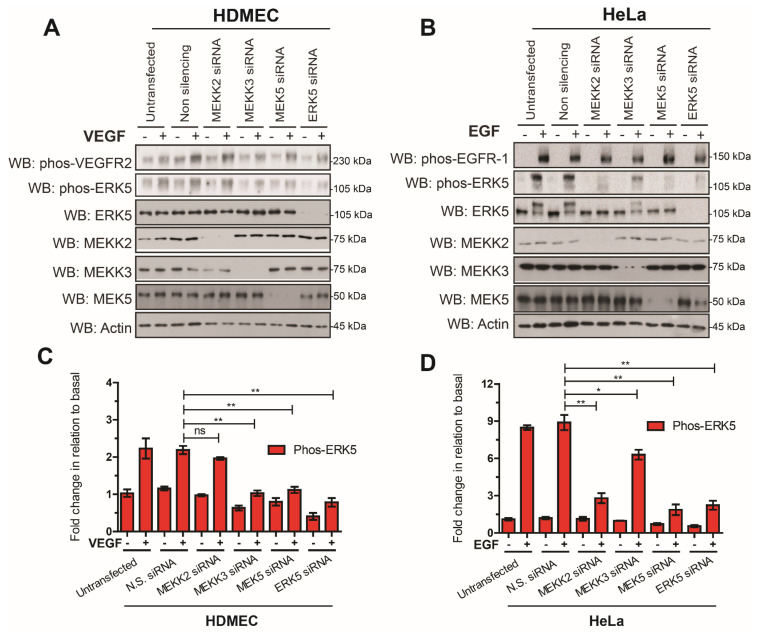
**Analysis of the canonical ERK5 signalling axis in HDMECs and HeLa cells.** (**A**) HDMECs or (**B**) HeLa cells were transfected with siRNA duplexes (10 nM) targeting MEKK2, MEKK3, MEK5, and ERK5 or non-silencing (N.S.) siRNA. Then, 24 h after transfection, cells were serum-starved overnight and then stimulated with VEGF (50 ng/mL) or EGF (50 ng/mL) for 10 min. Cells were lysed in RIPA buffer and lysates were resolved by SDS-PAGE and analysed by Western blotting. (**C**,**D**) Quantification of phospho-ERK5 (Thr218/Tyr220) level by densitometric analysis. Data are shown as mean fold change in relation to basal (untransfected cells) normalised to Actin level ± SEM (*n* = 3). Statistical analysis: one-way ANOVA followed by Tukey’s post hoc test, where * *p* < 0.05 and ** *p* < 0.01, ns = not significant.

ERK5 is activated by a linear signaling cascade consisting of MEKK2 and MEKK3, which phosphorylate MEK5, which, in turn, is able to phosphorylate ERK5 [17,18,31]. In order to confirm that ERK5 was activated by the canonical pathway in both HeLa and HDMECs, we utilized siRNA-mediated gene silencing of MEKK2, MEKK3, MEK5, and ERK5. Analysis of ERK5 phosphorylation revealed that, in HeLa cells, MEKK2 and MEK5 were required for EGF-stimulated ERK5 phosphorylation, whilst in HDMECs, MEKK3 and MEK5 were required for VEGF-stimulated ERK5 phosphorylation (Figure 3).

### 3.2. VEGFR-2 Activation Suppresses ERK5 C-Terminal Phosphorylation in Endothelial Cells

**Figure 4:**
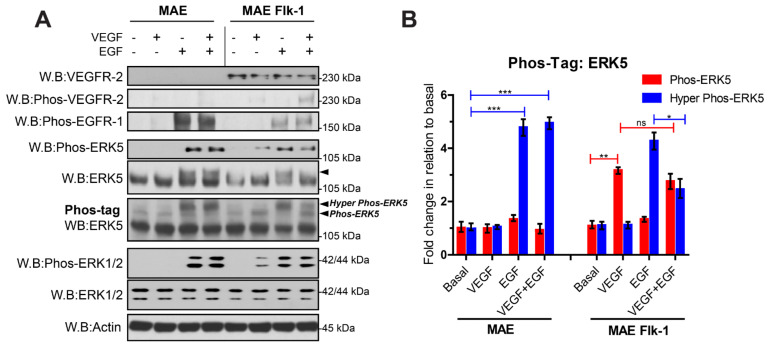
**Analysis of EGFR-1- and VEGFR-2-mediated ERK5 activation in murine endothelial cells.** (**A**) Murine Aortic Endothelial (MAE) cells or MAE-expressing Flk-1 were serum-starved overnight and stimulated with either VEGF (50 ng/mL), EGF (50 ng/mL) or both VEGF (50 ng/mL) + EGF (50 ng/mL) for 10 min. Cells were lysed in RIPA buffer, and lysates were resolved by SDS-PAGE or Phos-tag SDS-PAGE and analysed by Western blotting. (**B**) Quantification of ERK5 level by densitometric analysis. Data are shown as mean fold change relative to basal normalised to Actin level ± SEM (*n* = 3). Statistical analysis: one-way ANOVA followed by Tukey’s post hoc test, where * *p* < 0.05, ** *p* < 0.01, *** *p* < 0.001, ns = not significant.

Our data suggest that, in endothelial cells, VEGFR-2 activation of MEKK3/MEK5/ERK5 does not result in the C-terminal phosphorylation of ERK5. Primary human endothelial cells, such as HDMECs, are known to lack expression of EGFR-1 [37], which precluded a direct comparison of VEGF and EGF stimulation in these cells. However, murine aortic endothelial (MAE) cells express endogenous EGFR-1. We utilized the MAE Flk-1 cells stably expressing the murine VEGFR-2 to compare activation of EGFR-1 and VEGFR-2 in the same endothelial cell line (Figure 4). Analysis of proteins on Phos-tag acrylamide gels followed by blotting for ERK5 revealed that VEGF induced an increase in a band at approximately 115 kDa, similar to VEGF stimulation of HDMECs. In contrast, EGF stimulation resulted in the appearance of a higher migrating band at 125 kDa, similar to EGF stimulation of HeLa cells. Co-stimulation of the MAE Flk-1 cells with both VEGF and EGF enabled the two ERK5 phosphorylation events to be distinguished from one another within the same cell. Interestingly, it appeared that VEGF suppressed the appearance of the higher molecular weight band at 125 kDa stimulated by EGF; this effect was also evident on the conventional SDS-PAGE gel blotted for ERK5 (Figure 4).

### 3.3. VEGF-Stimulated ERK5 Regulates AKT Activity in HDMECs

**Figure 5:**
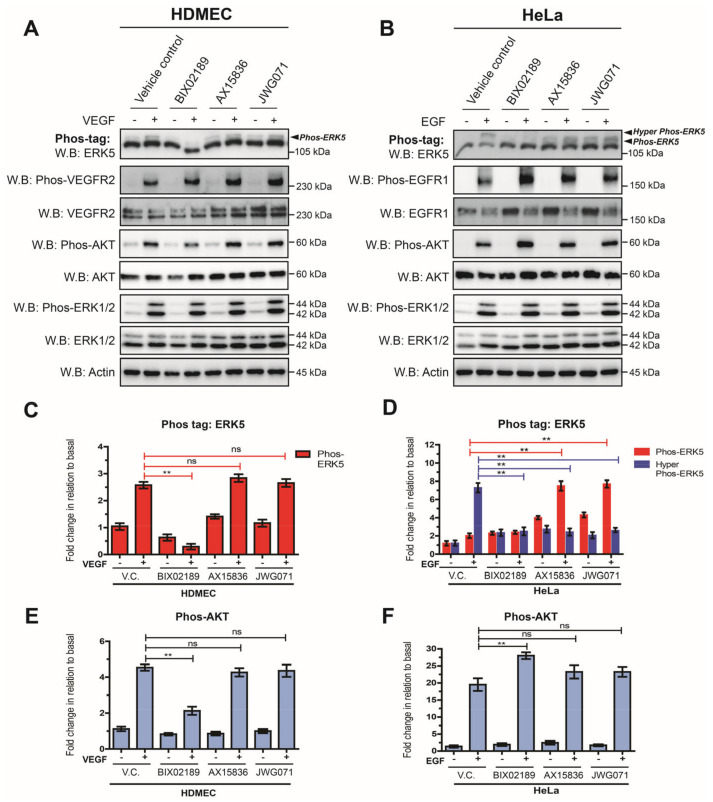
**VEGF-mediated ERK5 activation regulates AKT phosphorylation in endothelial cells.** (**A**) HDMECs or (**B**) HeLa cells were serum-starved overnight and then treated with either vehicle control (V.C.; 0.1% DMSO), or the MEK5 inhibitor BIX02189 (1 μM), or the ERK5 inhibitors AX15836 (1 μM) or JWG071 (1 μM) for 1 h prior to stimulation with either VEGF (50 ng/mL) or EGF (50 ng/mL) for 10 min. Cells were lysed in RIPA buffer and lysates were resolved by 10% SDS-PAGE or Phos-tag SDS-PAGE and analysed by Western blotting. (**C**) Quantification of HDMECs Phos-tag ERK5 level by densitometric analysis. Data are shown as mean fold change relative to basal (vehicle control) normalised to Actin level ± SEM (*n* = 3). (**D**) Quantification of HeLa Phos-tag ERK5 level by densitometric analysis. Data are shown as mean fold change relative to basal (vehicle control) normalised to Actin level ± SEM (*n* = 3). (**E**) Quantification of HDMECs Phos-AKT level by densitometric analysis. Data are shown as mean fold change relative to basal (vehicle control) normalised to Actin level ± SEM (*n* = 3). (**F**) Quantification of HeLa Phos-AKT level by densitometric analysis. Data are shown as mean fold change relative to basal (vehicle control) normalised to Actin level ± SEM (*n* = 3). Statistical analysis: one-way ANOVA followed by Tukey’s post hoc test, where ** *p* < 0.01, ns = not significant.

The apparent lack of C-terminal phosphorylation of ERK5 following VEGF stimulation in HDMECs suggested that VEGF/VEGFR-2 may couple to ERK5 in a novel way. Using siRNA-mediated gene silencing of ERK5, we have previously shown that VEGF-mediated ERK5 activity is required for efficient AKT phosphorylation in HDMECs [28]. In order to interrogate the role of MEK5 and ERK5 in regulating AKT activity further, we utilized small-molecule inhibitors to MEK5 kinase (BIX02189; [38]) and ERK5 kinase (AX15836; [39]) (JWG071; [40]) to probe the MEK5/ERK5 signalling axis in HDMECs and HeLa cells. In HDMECs, VEGF stimulated the appearance of a single ERK5 phosphoprotein band at 115 kDa when analysed on a Phos-tag gel. ERK5 band shift was prevented by pre-incubation with the MEK5 inhibitor BIX 02189, but not by the ERK5 kinase inhibitors AX15836 and JWG071 (Figure 5A).

Analysis of AKT phosphorylation revealed that BIX02189 was able to partly inhibit VEGF-mediated AKT phosphorylation by approximately 50% (Figure 5E). In contrast, the ERK5 kinase inhibitors AX15836 and JWG071 had no effect on VEGF-mediated AKT phosphorylation, indicating that ERK5 kinase activity was not required for activation of AKT. All inhibitors showed no apparent effect on VEGF-mediated ERK1/2 phosphorylation. In HeLa cells, pre-incubation with the MEK5 inhibitor BIX02189 prevented EGF-mediated ERK5 band shift on a Phos-tag gel (Figure 5B). Pre-incubation with the ERK5 inhibitors, AX15836 and JWG071, resulted in an ERK5 band shift, but not to the same degree as in the vehicle control; the hyper-phosphorylated form of ERK5 was not evident. No effect on ERK1/2 phosphorylation was evident with inhibitors. Analysis of AKT phosphorylation revealed that BIX02189 increased AKT phosphorylation, with no effect evident with AX15836 and JWG071 (Figure 5F). Taken together, these data show that in endothelial cells, VEGF/VEGFR-2-mediated activation of AKT is partly dependent on MEK5 kinase activity but not ERK5 kinase activity.

### 3.4. VEGF-Stimulated ERK5 Does Not Undergo Nuclear Translocation and Co-Localises with Phosphorylated AKT

Under basal conditions, ERK5 exists in a folded conformation in the cytoplasm via interaction of the N- and C-terminal domains. Agonist stimulation results in MEK-5-dependent phosphorylation of ERK5 on Thr218/Tyr220 and subsequent autophosphorylation of ERK5 on the C-terminal domain, resulting in nuclear translocation and ultimately transcriptional activity [23]. We were interested in determining if an apparent lack of VEGF-mediated C-terminal phosphorylation of ERK5 affects its translocation and possible co-localisation with phosphorylated AKT.

**Figure 6:**
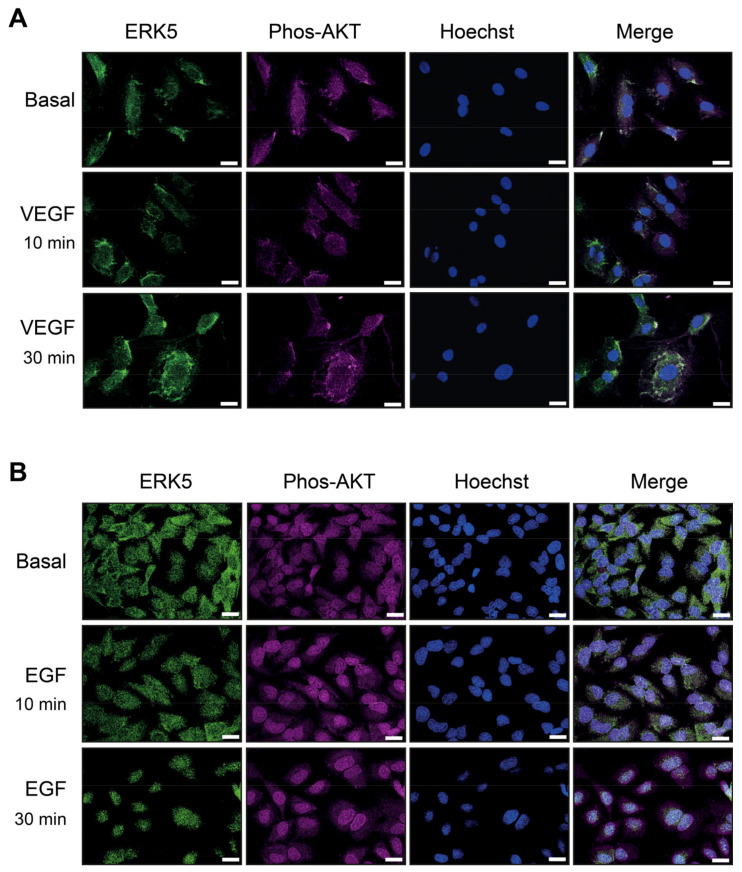
**VEGF stimulation results in cytosolic and plasma membrane localised ERK5, which co-localises with phosphorylated AKT in endothelial cells**. (**A**) HDMECs or (**B**) HeLa cells were serum-starved overnight and then treated with media (Basal) or stimulated with either VEGF (50 ng/mL) or EGF (50 ng/mL) for 10 min or 30 min. Cells were then fixed in paraformaldehyde, permeabilised, and stained with an antibody to ERK5 (green), phospho-AKT Ser373 (magenta) or Hoechst (blue) and analysed by immunofluorescence. Areas of pixel intensity correlation are shown as white/grey in the merged images. Scale bar = 10 μm. Results are from one experiment representative of three independent experiments.

HDMECs and HeLa cells were stimulated with VEGF and EGF, respectively, for 10 min and 30 min, followed by immunofluorescence analysis with an antibody to ERK5 and phosphorylated AKT. In HDMECs, ERK5 displayed diffuse staining under basal conditions. VEGF activation resulted in punctate staining around the cytoplasm and plasma membrane but no apparent nuclear localization (Figure 6A). It was not possible to directly analyse ERK5 phosphorylation by immunofluorescence due to cross-reaction of the phospho-ERK5 antibody (Thr218/Tyr220) with phosphorylated ERK1/2 (Thr202/Tyr204). Upon stimulation with VEGF, immunofluorescence analysis showed an apparent co-localisation of phosphorylated AKT with ERK5 (Figure 6A).

In unstimulated HeLa cells, diffuse ERK5 staining was observed throughout the cytoplasm. Upon EGF stimulation, ERK5 appeared to translocate into the nucleus by 10 min, and this nuclear localisation was sustained at 30 min stimulation (Figure 6B). Analysis of AKT phosphorylation revealed a nuclear localization under basal conditions with no apparent translocation upon EGF stimulation.

### 3.5. VEGF-Stimulated ERK5 Activity Does Not Regulate MEF2-Dependent Gene Expression in HDMECs

**Figure 7:**
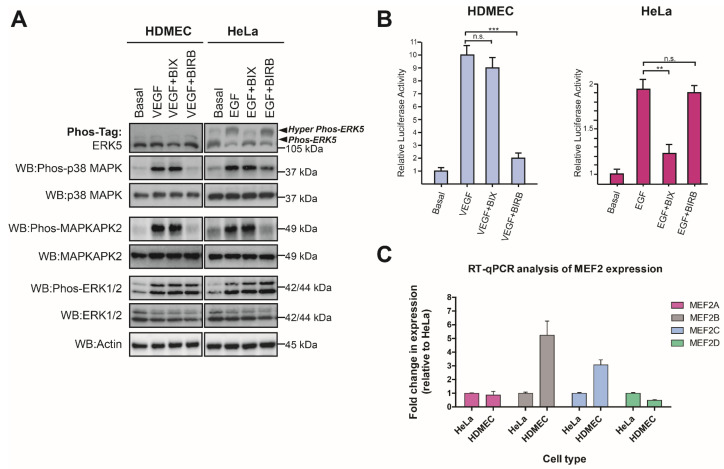
**VEGF-mediated ERK5 activity is not required for MEF2 transcriptional activity in endothelial cells.** (**A**) HDMECs and HeLa cells were serum-starved overnight and then treated with either vehicle control (0.1% DMSO) or the MEK5 inhibitor BIX02189 (BIX; 1 μM) or the p38 MAPK inhibitor BIRB796 (BIRB; 1 μM), for 1 h prior to stimulation with VEGF (50 ng/mL) or EGF (50 ng/mL) for 10 min. Cells were lysed in RIPA buffer, and lysates were resolved by 10% SDS-PAGE or Phos-tag SDS-PAGE and analysed by Western blotting. (**B**) HDMECs and HeLa cells were transduced with Ad-MEF2-Luc (MOI 50) and Ad-CMV-LacZ (MOI 20) for 24 h prior to serum starvation overnight. Cells were treated with either vehicle control (0.1% DMSO) or the MEK5 inhibitor BIX02189 (BIX; 1 μM) or the p38 MAPK inhibitor BIRB796 (BIRB; 1 μM), for 1 h prior to stimulation with VEGF (50 ng/mL) or EGF (50 ng/mL) for 6 h. Cells were lysed and MEF2 reporter activity determined by luciferase activity. Relative luciferase activities were normalised to beta galactosidase activity and compared to basal response ± SEM (*n* = 3). (**C**) Analysis of *MEF2* family mRNA levels by qRT-PCR in HDMECs and HeLa cells. Expression levels of *MEF2A*, *MEF2B*, *MEF2C,* and *MEF2D* were determined by qRT-PCR and normalised to *β-ACTIN* level. Results are plotted as fold change relative to level in HeLa cells ± range (*n* = 2). Statistical analysis: one-way ANOVA followed by Tukey’s post hoc test, where ** *p* < 0.01, *** *p* < 0.001, ns = not significant.

Translocation of ERK5 to the nucleus results in the phosphorylation and activation of the MEF2 family of transcription factors [12,41]. In addition to ERK5, the p38 MAPK family has also been shown to regulate MEF2 phosphorylation and transcriptional activity [42]. Both VEGF stimulation of HDMECs and EGF stimulation of HeLa cells resulted in activation of p38MAPK and increased phosphorylation of the specific p38 MAPK substrate MAPK-activated protein kinase 2 (MAPKAPK2) [43] (Figure 7A). The inability of VEGF-activated ERK5 to translocate to the nucleus in endothelial cells suggested that ERK5 may play a different role in endothelial cells, compared with the canonical role in regulating MEF2-dependent gene expression in HeLa cells. We used adenoviral-mediated expression of a MEF2-Luc reporter to analyse activation of MEF2 family proteins and their subsequent ability to stimulate transcription in VEGF-stimulated HDMECs and EGF-stimulated HeLa cells. VEGF induced an increase in MEF2 transcriptional activity, which was not blocked by the MEK5 inhibitor BIX02189 but was inhibited by the p38 MAPK inhibitor BIRB796 [44] (Figure 7B). In contrast, in HeLa cells, EGF-stimulated MEF2 transcriptional activity was partially inhibited by MEK5 inhibitor BIX02189, with no apparent effect with the p38 MAPK inhibitor BIRB796 (Figure 7B). Analysis of mRNA expression of MEF2 isoforms in the HeLa cells and HDMECs revealed that both cells have comparable levels of *MEF2A* and *MEF2D*, whereas the HDMECs showed higher expression of *MEF2B* and *MEF2C*. These data show that in HDMECs, VEGF-stimulated ERK5 activity does not appear to regulate MEF2 transcriptional activity.

## 4. Discussion

Gene ablation in mice has shown that ERK5 is critical for embryonic development with *Erk5*^−/−^ mice displaying defects in cardiovascular development [45]. Additionally, endothelial-specific *Erk5*-knockout leads to cardiovascular defects identical to that of global *Erk5*-knockout mutants [27]. These data suggest that whilst ERK5 is expressed in many different cell types, it plays a unique role in endothelial cells compared to other cells. We have previously shown that ERK5 is critical for VEGF-mediated AKT activation in HDMECs and tubular morphogenesis [28]. Building on these previous data, our present study shows that in HDMECs, the VEGFR-2 couples to ERK5 activation via a mechanism that results in ERK5 activation in the absence of C-terminal phosphorylation. ERK5 translocates to the cytoplasm and plasma membrane and co-localises with AKT resulting in the regulation of AKT phosphorylation. This is in contrast to the canonical pathway of ERK5 activation, resulting in C-terminal phosphorylation and translocation to the nucleus and concomitant increase in MEF2 transcriptional activity, as was observed for HeLa cells stimulated with EGF. Our data confirm a unique role for ERK5 in endothelial cell physiology.

Agonist-mediated activation of ERK5 involves dual phosphorylation of the TEY motif within the activation loop of the kinase domain, a process catalyzed by the upstream kinase MEK5 [8,46]. This ultimately results in the C-terminal autophosphorylation of ERK5 on multiple serine and threonine residues and nuclear localization. Phosphorylation of the TEY motif followed by C-terminal phosphorylation were thought to be mutually inclusive events. However, our data show that whilst VEGF-mediated ERK5 activation was detectable on the TEY motif, C-terminal phosphorylation could not be detected. The VEGF/VEGFR-2 pathway leading to ERK5 phosphorylation on the TEY motif proceeded via the MEK5 pathway, similar to EGF/EGFR-1 pathway in HeLa cells (Figure 3 and Figure 5). The two pathways, however, deviated at the level of the MAPKKK, with VEGF/VEGFR-2 specifically utilising MEKK3 and EGF/EGFR-1 preferentially utilising MEKK2 (Figure 3) to activate MEK5 and ultimately allow phosphorylation of ERK5 on the TEY motif. This is despite both cell types expressing MEKK2 and MEKK3 proteins (Figure 3). It is possible that a potential association between the ligand-activated VEGFR-2 and MEKK3 in the endothelial cells prevents, or fails, to facilitate C-terminal phosphorylation of ERK5, whilst EGFR-1 association with MEKK2 facilitates efficient phosphorylation of ERK5. In terms of EGFR-1 signalling, MEKK2 has been shown to bind to the adaptor protein Lad facilitating Src-mediated phosphorylation of Lad and activation of ERK5; interestingly, the authors reported that MEKK3 did not bind to Lad [47].

Another pathway that may allow a differential C-terminal phosphorylation of ERK5 by different receptor tyrosine kinases (RTKs) could involve a MEK5-independent route through the involvement of other kinases, as has been shown for ERK5 during mitosis in HeLa cells [36]. Whilst our data using the ERK5 kinase inhibitors confirm a role for the ERK5 kinase in C-terminal phosphorylation of ERK5 in HeLa cells (Figure 4), it is possible that ancillary pathways and kinases, not engaged by VEGFR-2, are responsible for priming the ERK5 C-terminus facilitating more efficient phosphorylation by the kinase domain. Recent data have pointed to a role for ERK1/2 in phosphorylating ERK5 on Thr732 in the C-terminal domain [48]. It is unlikely that this mechanism can explain the differential phosphorylation of ERK5 on Thr732, as both VEGF stimulation in HDMECs and EGF stimulation in HeLa evoked a robust activation of ERK1/2 (Figure 1 and Figure 5).

Nuclear translocation of ERK5 requires both a conformational change following phosphorylation of the TEY motif by MEK5 and C-terminal phosphorylation by the kinase domain, allowing exposure of the nuclear NLS [23,49]. In HeLa cells, it has been reported that ERK5 nuclear translocation requires dissociation of Hsp90 from a cytosolic ERK5-Cdc37 complex [50]. The inability of VEGF-VEGFR-2 activation to induce C-terminal phosphorylation of ERK5 in HDMECs may result in a lack of Hsp90 release and nuclear entry. A role for ERK5 in regulating AKT phosphorylation in response to VEGF in HDMECs has been previously shown by our group [28], a pathway critical for endothelial cell survival. Use of MEK5 and ERK5 inhibitors showed that VEGF/VEGFR-2-mediated MEK5 phosphorylation of the TEY motif was critical for regulating AKT activity, whilst inhibition of ERK5 kinase activity did not affect AKT phosphorylation (Figure 5), consistent with the fact that C-terminal phosphorylation is not occurring. We are now able to show apparent co-localisation of ERK5 and phosphorylated AKT in a cytoplasm/plasma membrane area following VEGF stimulation of HDMECs (Figure 6). Interestingly, EGF stimulation of HeLa cells also showed an apparent co-localisation of ERK5 and phosphorylated AKT in the nucleus. It is tempting to speculate that this close association of ERK5 and AKT in different intracellular locations may suggest a potential chaperone role for ERK5. However, the precise mechanism of ERK5-mediated AKT phosphorylation in endothelial cells remains obscure.

Our data showed that whilst ERK5 did not appear to regulate MEF2 transcriptional activity following VEGF stimulation in endothelial cells, p38 MAPK was responsible for facilitating VEGFR-2-mediated MEF2 activity. This was in contrast to EGFR-1, where ERK5 activity was required for EGFR-1-mediated MEF2 activity, in agreement with the previously reported role of ERK5 in regulating EGF-stimulated MEF2 activity in HeLa cells [12]. A previous study utilizing overexpression of dominant-negative ERK5 and dominant-negative p38 MAPKα in retinal endothelial cells also reported that ERK5 was not required for MEF2-dependent transcription, in contrast to p38 MAPKα [51]. The inability of VEGFR-2 activation to promote a nuclear localization of ERK5 (Figure 6) is a potential explanation for the ERK5-independent MEF2 activity observed (Figure 7).

**Figure 8:**
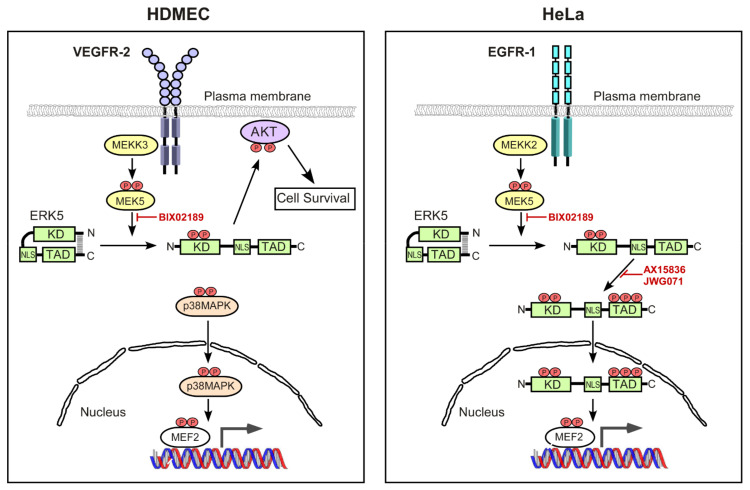
**Schematic diagram of differential ERK5 activation by VEGF in endothelial cells and EGF in HeLa cells.** Ligand-mediated activation of EGFR-1 in HeLa cells activates MEK5 predominantly via MEKK2, resulting in the phosphorylation of ERK5 on the activation-loop T-E-Y motif in the ERK5 kinase domain, resulting in a conformational change in ERK5 and facilitating phosphorylation of ERK5 on multiple residues in the C-terminal transcriptional transactivation domain (TAD). The nuclear localisation sequence (NLS) within the C-terminal domain is now able to promote a nuclear localisation of ERK5. ERK5 is thought to drive MEF2-dependent gene expression by both directly phosphorylating MEF2 transcription factors and by the involvement of the ERK5 TAD. In contrast, ligand-mediated activation of VEGFR-2 in endothelial cells activates MEK5 via MEKK3, resulting in the phosphorylation of ERK5 on the activation-loop T-E-Y motif in the ERK5 kinase domain. However, ERK5 does not undergo phosphorylation in the C-terminus and is unable to translocate to the nucleus, and is instead localised to the cytoplasmic and plasma membrane area ultimately allowing phosphorylation of AKT and suppression of apoptosis in endothelial cells. In contrast to EGFR-1 signalling, MEF2 transcriptional activity in response to VEGFR-2 activation proceeds via p38 MAPK.

Gene knockout studies in mice have revealed a fundamental role for the ERK5 signalling axis in vascular function. Gene knockout of *Mekk3*, *Mek5,* and *Erk5* results in embryonic lethality at E10.5-11, with defects in angiogenesis and endothelial cell development [16,24,25,26,52,53]. Interestingly, gene knockout of *Mekk2* has no discernable effect as mice develop normally [54,55,56], which supports our observation that in contrast to MEKK3, MEKK2 does not appear to regulate VEGFR-2-mediated ERK5 activity in endothelial cells. The requirement for ERK5 activity to protect endothelial cells from apoptosis has been ascribed to the need for ERK5-mediated phosphorylation of the MEF2C transcription factor [27]. This is based on the fact that the phenotype of *Mef2c*^–/–^ mice is similar to that of *Erk5*^–/–^ mice, with embryonic lethality resulting from cardiac and vascular malformations [27,45]. However, infection of *Erk5*^–/–^ embryos with an adenovirus encoding a constitutively active Mef2c was only able to partially protect endothelial cells from apoptosis [27], suggesting the existence of additional effectors downstream of ERK5 that regulate apoptosis [57]. Our data show a role for ERK5 in regulating AKT activity, rather than MEF2 activity, in endothelial cells (Figure 8). Interestingly, gene knockout of *Akt1* and *Akt3* results in embryonic lethality with mice displaying cardiac and vascular defects [58]; VEGFR-2/ERK5/AKT may represent a key signalling pathway for endothelial cell survival during embryonic development.

Our data define a novel mechanism of VEGF/VEGFR-2-mediated activation of ERK5 in endothelial cells, resulting in the activation of AKT, which differs from the previously assumed canonical pathway based on EGFR-1 activation of ERK5 (Figure 8). Further delineation of the mechanism of ERK5-mediated AKT activation will allow us to gain a better understanding of the key role of the ERK5 signalling axis in vascular development and maintenance.

## Data Availability

Data are available upon request. Please contact corresponding author M.J.C.

## Supplementary Materials

The following supporting information can be downloaded at: https://www.mdpi.com/article/10.3390/cells12060967/s1, Figure S1: VEGF and EGF stimulate differential ERK5 activation in human endothelial cells and HeLa cells, respectively.
