# Supplementary material for: VEGF Stimulates Activation of ERK5 in the Absence of C-Terminal Phosphorylation Preventing Nuclear Localization and Facilitating AKT Activation in Endothelial Cells
Source: Cells. 2023 Mar 22;12(6):967. doi: 10.3390/cells12060967 (PMC10047687; doi:10.3390/cells12060967)
Supplement: Supplementary file 1 [file cells-12-00967-s001.zip › cells-2225965-supplementary.pdf]

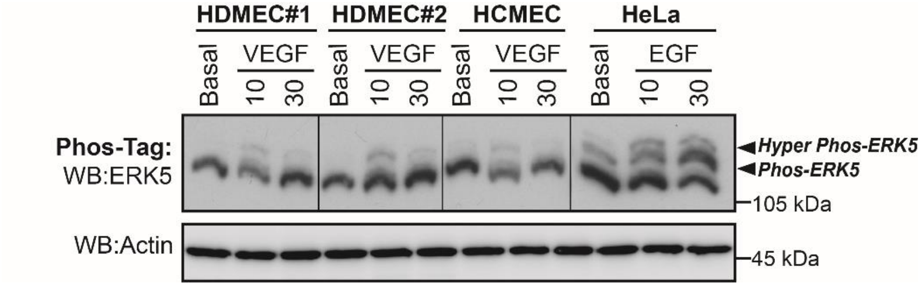

Figure S1. VEGF and EGF stimulate differential ERK5 phosphorylation. Human dermal microvascular endothelial cells (HDMECs) from two different donors and human cardiac microvascular endothelial cells (HCMECs) were serum starved overnight prior to stimulation for 10 mins with VEGF (50ng/ml). HeLa cells were serum starved overnight prior to stimulation for 10 mins with EGF (50ng/ml). Cells were lysed in RIPA buffer and lysates were resolved by 10% SDS-PAGE (Actin) or Phos-tag SDS-PAGE (ERK5) and analysed by western blotting. Results are from one experiment representative of two independent experiments.
